# The Impact of Uranium-Induced Pulmonary Fibrosis on Gut Microbiota and Related Metabolites in Rats

**DOI:** 10.3390/metabo15080492

**Published:** 2025-07-22

**Authors:** Ruifeng Dong, Xiaona Gu, Lixia Su, Qingdong Wu, Yufu Tang, Hongying Liang, Xiangming Xue, Teng Zhang, Jingming Zhan

**Affiliations:** 1 Key Laboratory of Radiation Environment & Health of the Ministry of Ecology and Environment, China Institute of Radiation Protection, Taiyuan 030006, China; ruifengdong@126.com (R.D.); guxiaona6954@163.com (X.G.); sulixia407@163.com (L.S.); axiwqd@163.com (Q.W.); tangyufu2022@163.com (Y.T.); lianghongying0218@163.com (H.L.); zyws0351@163.com (X.X.); 2 Shanxi Key Laboratory of Forensic Medicine, School of Forensic Medicine, Shanxi Medical University, Jinzhong 030605, China

**Keywords:** uranium-induced pulmonary fibrosis, gut microbiota, metabolomics, gut–lung axis

## Abstract

**Background/Objectives**: This study aimed to evaluate the effects of lung injury induced by insoluble uranium oxide particles on gut microbiota and related metabolites in rats. **Methods**: The rats were randomly divided into six UO_2_ dose groups. A rat lung injury model was established through UO_2_ aerosol. The levels of uranium in lung tissues were detected by ICP-MS. The expression levels of the inflammatory factors and fibrosis indexes were measured by enzyme-linked immunosorbent assay. Paraffin embedding-based hematoxylin & eosin staining for the lung tissue was performed to observe the histopathological imaging features. Metagenomic sequencing technology and HM700-targeted metabolomics were conducted in lung tissues. **Results**: Uranium levels in the lung tissues increased with dose increase. The expression levels of Tumor Necrosis Factor-α (TNF-α), Interleukin-1β (IL-1β), Collagen I, and Hydroxyproline (Hyp) in rat lung homogenate increased with dose increase. Inflammatory cell infiltration and the deposition of extracellular matrix were observed in rat lung tissue post-exposure. Compared to the control group, the ratio of *Firmicutes* and *Bacteroides* in the gut microbiota decreased, the relative abundance of *Akkermansia_mucinphila* decreased, and the relative abundance of *Bacteroides* increased. The important differential metabolites mainly include αlpha-linolenic acid, gamma-linolenic acid, 2-Hydroxybutyric acid, Beta-Alanine, Maleic acid, Hyocholic acid, L-Lysine, L-Methionine, L-Leucine, which were mainly concentrated in unsaturated fatty acid biosynthesis, propionic acid metabolism, aminoacyl-tRNA biosynthesis, phenylalanine metabolism, and other pathways in the UO_2_ group compared to the control group. **Conclusions**: These findings suggest that uranium-induced lung injury can cause the disturbance of gut microbiota and its metabolites in rats, and these changes are mainly caused by *Akkermansia_mucinphila* and *Bacteroides*, focusing on unsaturated fatty acid biosynthesis and the propionic acid metabolism pathway.

## 1. Introduction

Inhalation exposure to insoluble uranium particles is the most predominant form of exposure in the population, especially in the occupational population [1]. When exposed through inhalation, insoluble particles can linger in lung tissue and nearby lymph nodes for a long time, leading to pulmonary fibrosis [2,3,4]. Pulmonary fibrosis (PF) is a chronic inflammatory disease characterized by abnormal proliferation of fibroblasts, excessive deposition of extracellular matrix proteins, accompanied by inflammatory damage, which steadily leads to lung architecture disruption and respiratory failure [5]. Despite extensive research that has been conducted in understanding the molecular mechanism of PF, the effective approaches for pulmonary fibrosis early diagnosis and treatment are currently lacking. How to delay or even reverse its progression has been a difficult problem in the field of respiratory diseases [6,7,8].

Patients with chronic lung diseases such as respiratory tract infection, asthma, and pulmonary fibrosis are often accompanied by intestinal symptoms, and the degree of intestinal damage is highly consistent with the severity of lung lesions, and there is a regulatory network or information exchange system for the gut–lung axis [9,10]. The regulation function of the gut–lung axis is reflected in three aspects: microorganisms, microbial metabolites, and immune function regulation [11]. First, the gut microbiota consists of millions of microorganisms that colonize the intestinal mucosal barrier and participate in a variety of processes, such as immune modulation, energy homeostasis, and lipid and carbohydrate metabolism [12,13,14]. The microecological disturbance caused by changes in the composition of the intestinal flora can affect the course of pulmonary fibrosis [15]. Animal experiments [16,17,18,19,20] and population studies [21,22] have shown that even subtle changes in the composition of gut microbiota can ripple through the body, influencing the progression of pulmonary fibrosis. Second, gut microbes produce a diverse array of functional metabolites and small molecules, such as short-chain fatty acids [23,24], tryptophan derivatives [25], vitamins [26], bile acids [27,28], and polyphenol metabolites [29]. These microbial products can be subsequently absorbed by the host intestine and exert multifaceted effects on pulmonary inflammatory states [30,31,32]. Third, the gut microbiota is indispensable for the development and regulation of the host immune system. The imbalance of gut microbiota not only regulates the immune response of the gastrointestinal tract, but also affects the immunity of distant organs (such as the lung). This gut–lung immune axis plays a significant role in lung homeostasis and the pathophysiology of respiratory diseases, including pulmonary fibrosis [33].

This present study aims to investigate the effects of lung injury induced by insoluble uranium oxides on gut microbiota and related metabolites in rats from the perspective of “lung–intestine” by means of metagenomics and metabolomics. It also investigates the potential mechanisms of uranium-induced lung injury and offers new ideas for early diagnosis and prevention strategies.

## 2. Materials and Methods

### 2.1. Animal, Fibrotic Models, and Sample Collection

SPF SD male rats (about 9 to 10 weeks of age) were purchased from Beijing Weitonglihua Experimental Animal Technology Co., Ltd., Beijing, China [SCXK (Beijing) 2021-0011], then housed under standard vivarium conditions for 1 week.

All rats were randomly assigned to 6 dose groups with 8 rats in each group, the rats were injected intraperitoneally and anesthetized suitably with 1% pentobarbital sodium, and then vertically mounted on a slope of cystosepiment. A laryngoscope was held at the throat of the rat and the tongue was pulled gently, a rat lung dry powder quantitative nebulizer containing UO_2_ powder (administering doses of 0, 0.5, 1, 2, 4, 8 mg, respectively) was inserted into the rat trachea, and the syringe piston was quickly pressed to the end.

The fecal pellets were collected by using metabolism cage from 1st, 7th, and 14th day after exposure to UO_2_, which were used for the determination of uranium content in feces. The cecal contents of rats in 0, 0.5, 2, 8 mg dose groups were collected from 7th day after exposure to UO_2_, which were used for metagenomic sequencing and HM700 targeted metabolomics studies (the 0.5, 2, and 8 mg dose groups represent the low-dose, medium-dose, and high-dose groups, respectively).

### 2.2. Pathological Analysis and Hydroxyproline Quantification

Rats were anesthetized deeply with pentobarbital sodium, and killed by excessive loss of blood after the abdominal aorta were cut off, then 4% paraformaldehyde-fixed tissues were routinely processed, embedded in paraffin, sectioned at 5 μm. The slices were stained with HE; the stained slides were photographed by a fluorescence microscopy imaging system (Olympus Corporation, Yoshino-shi, Japan). The whole low-right lobe was used for hydroxyproline acid measurement by using Hydroxyproline assay kit (Nanjing Jiancheng, Nanjing, China).

### 2.3. Metagenomics Sequencing

Total genome DNA was extracted from fecal pellets by using CTAB/SDS method. According to the specific requirements of the samples and products, appropriate detection methods were selected for quality assessment. The concentration (≥12.5 μg/μL), integrity (main peak in the electrophoretic gel profile >20 kb), and purity (free from protein, RNA, or salt ion contamination) were rigorously evaluated.

A certain amount of qualified metagenomic DNA was taken and then subjected to random fragmentation into 300~400 bp fragments using Covaris ultrasonic fragmentation. Subsequently, a series of steps, including end repair, A-tailing, sequencing adapter ligation, purification, PCR amplification, and product circularization, were performed to complete the library preparation process. Chained DNA molecules are replicated by rolling rings to form a DNA nanosphere (DNB) containing multiple copies. The DNBs were loaded into the mesh pores of a chip using high-density DNA nanoball technology, and sequenced using combined probe anchoring polymerization (cPAS) technology. DNBSEQ T7 platform was used for sequencing with PE150.

### 2.4. Gene Prediction and Abundance Information

MetaGeneMark (Version 3.38) was used to predict metagenomic genes from scratch, and then CD-HIT (Version 4.81) software was used to eliminate the redundancy of gene prediction results of each sample. According to the sequence similarity (set identity threshold of 95%, coverage threshold of 90%), they are classified into one of the categories or become the representative sequence of a new cluster, so that all sequences are traversed to complete the clustering process. Finally, Salmon (Version 1.60) software was used for quantitative analysis, and the TPM value obtained was the standardized gene abundance value.

### 2.5. LefSe Analysis of Different Gut Microbiota

Linear discriminant analysis Effect Size (LEfSe) can directly analyze the difference at all classification levels simultaneously, and can be used to find the species (called biological markers, biomarkers, or features) that best explain the difference between two or more groups of samples, and the extent to which these species contribute to inter-group differences (LDA). In this study, the taxa of species with LDA > 2 and *p* < 0.05 were considered to have statistical differences, focusing on species with differences in each group at the species level.

### 2.6. Targeted Metabolomics Profiling

UPLC-MS Analysis was accomplished by using the Waters ACQUITY UPLC I-Class Plus (Waters, Milford, MA, USA) Series QTRAP6500 Plus High Sensitivity Mass Spectrometer (SCIEX, Framingham, MA, USA). Samples were analyzed by using a BEH C18 (2.1 mm × 10 cm, 1.7 µm, waters) column for HILIC separation. The mobile phase contained A = 0.1% formic acid water and B = 30% isopropanol acetonitrile. The gradient was 0~1.00 min, 5% B; 1.00~5.00 min, 5~30% B; 5.00~9.00 min, 30~50% B; 9.00~11.00 min, 50~78% B; 11.00~13.50 min, 78~95% B; 13.5~14.00 min, 95~100% B; the above flow rate was 0.400 mL/min; 14.00~16.00 min, 100% B; the flow rate is 0.600 mL/min; 16.00~18.00 min, 5% B; flow rate was 0.400 mL/min, column temperature was 40 °C.

Mass spectrum (MS) conditions: For the QTRAP 6500 Plus (SCIEX, Framingham, MA, USA) equipped with ESI Turbo ion spray interface, Ion Source parameters are set as follows: Ion Source temperature: 400 °C; Ion spray voltage (IS): ±4500 V; Ion Source Gas I (GS1), Gas II (GS2), and Curtain Gas (CUR) as 60, 60, and 35 psi, respectively. The MRM method is set in MRM mode, which contains the MRM parent–child ion pair information of the target metabolite, the collision energy (CE) and declustering potential (DP), and retention time.

### 2.7. Analysis of Targeted Metabolomics Profiling Data

The raw MS was extracted and identified by using Quantitative software skyline (version: v.21.1.0.146); the peak area and identification results of metabolites were obtained. Orthogonal Partial Least-Squares Discrimination Analysis (OPLS-DA) wase performed after the Pareto scaling. The leave one which is out 7-fold cross-validation and response permutation testing was used to evaluate the robustness of the model. Differential metabolites were screened by three indicators: fold change (FC ≥ 1.2 or ≤0.83) by univariate analysis, significance level by multiple tests (Q-value < 0.05), and VIP value by multivariate analysis.

Differential metabolites were selected to perform for KEGG pathway analysis. Pathways of metabolites with a *p*-value < 0.05 were defined as those with significant enrichment of differential metabolites, bubble map and pathway score map of the top 10 metabolic pathways with the lowest *p*-value.

### 2.8. Statistical Analysis

The normal distribution of data was assessed using the Shapiro–Wilk test. All results excluding bioinformatics analysis were presented as mean ± SEM. Statistical analysis was conducted using one-way ANOVA in GraphPad Prism 9, followed by Tukey’s multiple comparisons test for post hoc analysis. Data correlation analysis was performed using Pearson’s correlation coefficient. *p* values of <0.05 were considered significant.

## 3. Results

### 3.1. Alterations of Uranium Content in Rat Tissue

**Figure 1:**
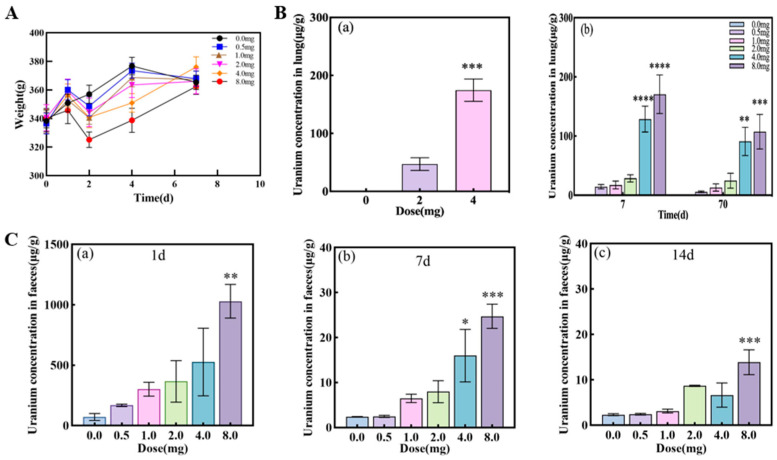
Changes in body weight and uranium levels in the lungs and feces of rats in different dose groups. (**A**) Body weight changes in different doses of UO_2_ for 1 week; (**B**) uranium levels in rat lungs at various time points post-exposure to different doses of UO_2_. (**a**) Uranium levels in rat lungs immediately killed post-exposure; (**b**) uranium levels in rat lungs at 7 and 70 days post-exposure to varying doses of UO_2_; (**C**) uranium levels in rat feces at various time points post-exposure to different doses of UO_2_. (**a**) 1 day post-exposure; (**b**) 7 days post-exposure; (**c**) 14 days post-exposure. The asterisks represent the significance levels compared to the control group, with *, **, ***, and **** representing *p* < 0.05, *p* < 0.01, *p* < 0.001, and *p* < 0.0001, respectively.

Compared to the control group, the body weight of rats in the UO_2_ group decreased significantly on the second day post-exposure but gradually recovered to normal levels thereafter. Notably, the 4 mg and 8 mg dose groups exhibited a more pronounced decline in body weight (Figure 1A). Following UO_2_ aerosol inhalation, rats were immediately dissected, and the lungs were excised to measure uranium content. The uranium concentration in the lungs of the control group (0.052 ± 0.018 μg/g) was significantly lower than that of the 2 mg dose group (47.13 ± 15.41 μg/g) and the 4 mg dose group (268.47 ± 174.57 μg/g). The difference in lung uranium content between the 4 mg UO_2_ group and the control group was statistically significant (*p* < 0.001). Uranium levels in the lungs increased with higher doses, but no significant temporal changes were observed post-exposure. The pulmonary deposition fraction ranged from 2.39% to 5.24% at 7 days post-exposure and 2.35% to 4.31% at 70 days post-exposure. By day 70, lung deposition retained 86% of the initial uranium load (Figure 1B). Additionally, uranium content in rat excrement increased significantly with higher UO_2_ doses, with the 8 mg dose group showing a statistically significant difference compared to the control group (*p* < 0.01). The uranium levels in feces decreased significantly over time following UO_2_ exposure (Figure 1C).

### 3.2. The Establishment of Pulmonary Fibrosis Rat Models Induced by UO_2_

**Figure 2:**
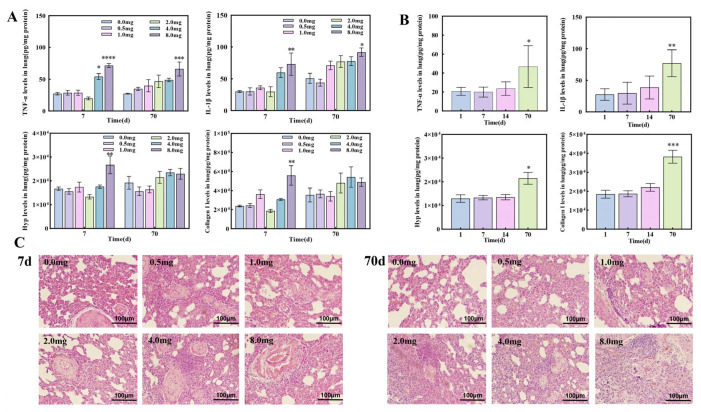
Changes in various indicators in rat lung homogenate and pathological tissue results of the lungs. (**A**). Contents of TNF-α, IL-1β, Collagen I, Hyp, Collagen I in lung homogenates of rats 7 and 70 days after exposure to UO_2_ at different doses. (**B**). Contents of TNF-α, IL-1β, Hyp, and Collagen I in lung homogenates of rats at different time points after 2 mg UO_2_ exposure. (**C**). HE staining of the lungs of rat groups 7 days and 70 days after exposure to different doses of UO_2_, magnification: HE × 200. The asterisks represent the significance levels compared to the control group or the first day after 2 mg UO_2_ exposure, with *, **, *** and **** representing *p* < 0.05, *p* < 0.01, *p* < 0.001 and *p* < 0.0001, respectively.

After exposure to UO_2_, a marked elevation in the expression levels of TNF-α, IL-1β, Collagen I, and Hydroxyproline (Hyp) was observed in the lung homogenate of rats at the 8 mg dosage (Figure 2A). As the post-exposure period extended to 70 days, there was a significant temporal increase in the expression levels of TNF-α, IL-1β, Collagen I, and Hyp within the lung homogenate of the rats (Figure 2B). HE staining revealed that the alveolar structure in the control group was well-defined, with uniform lung interstitial thickness and no significant inflammatory infiltration. In contrast, UO_2_-exposed rats exhibited markedly widened alveolar intervals accompanied by substantial inflammatory infiltrates. Ultimately, the alveolar interstitial collagen fibers were observed to thicken. The severity of lung injury in rats exposed to UO_2_ for 70 days was notably greater compared to those exposed for 7 days (Figure 2C).

### 3.3. Alterations of Rats’ Gut Microbiota upon Induction of Pulmonary Fibrosis

**Figure 3:**
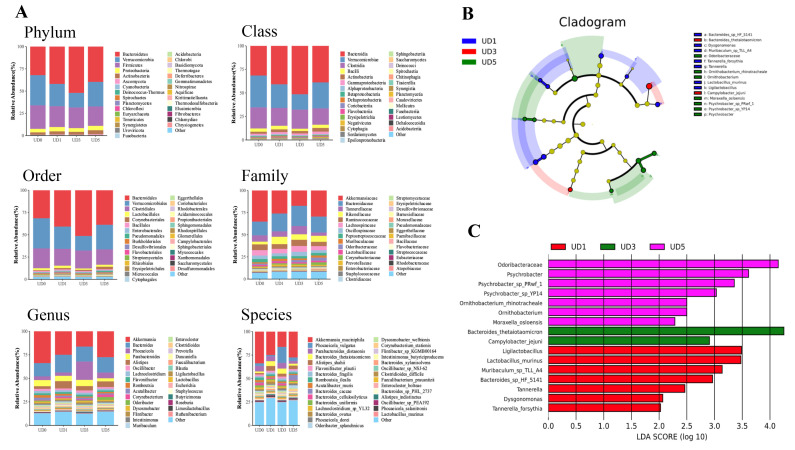
Alterations of rats’ gut microbiota. (**A**) Species relative abundance of intestinal contents of rats in different dose groups at phylum, class, order, family, genus, and species levels (n = 5). (**B**) LEfSe ring branching diagram. (**C**) Histogram of LEfSe LDA value distribution.

The species distribution of the microbial community represents fundamental information about its composition. Compared to the control group, the Chao 1 index in the 0.5 mg and 8 mg groups exhibited significant differences at the phylum level (*p* < 0.05). The richness of gut microbiota in the intestinal contents of rats across all dose groups decreased, while the evenness remained relatively unchanged. Notably, significant differences in gut microbiota structure were observed among the groups. The species distribution and compositional abundance of microbial communities in samples from each dose group were analyzed at the phylum, class, order, family, genus, and species levels (Figure 3A). At all taxonomic levels, significant alterations in the relative abundance of bacteria were observed in samples from different dose groups. These changes were primarily driven by variations in the abundance of *Akkermansia muciniphila* (AKK) and *Bacteroides*.

There were 16 different species after uranium-induced lung injury, among which *Bacteroides_thetaiotaomicron* had the highest score (LDA = 4.25) (Figure 3B). The gut microbiota of 0.5 mg rats combined with *Ligilactobacillus* (LDA = 3.49), *Lactobacillus_murinus* (LDA = 3.47), *Muribaculum_*sp.*_TLL_A4* (LDA = 3.14), *Bacteroides_*sp.*_HF_5141* (LDA = 2.96), *Tannerella* (LDA = 2.46), *Dysgonomonas* (LDA = 2.07), and *Tannerella_forsythia* (LDA = 2.03) increased significantly, which was the different species in the 0.5 mg group. The gut microbiota of the 2 mg group, *Bacteroides_thetaiotaomicron* (LDA = 4.25) and *Campylobacter_jejuni* (LDA = 2.91), had higher enrichment, which were the different species of the 2 mg group. The gut microbiota of the 8 mg group, the enrichment degree of *Odoribacteraceae* (LDA = 4.15), *Psychrobacter* (LDA = 3.61), *Psychrobacter_*sp.*_PRwf_1* (LDA = 3.36), *Psychrobacter_*sp.*_YP14* (LDA = 3.03), *Ornithobacterium_rhinotracheale* (LDA = 2.50), *Ornithobacterium* (LDA = 2.50), and *Moraxella_osloensis* (LDA = 2.28) were higher, which were the different species in the 8 mg group (Figure 3C).

### 3.4. Alterations of Metabolites in Enteric Canal of Pulmonary Fibrosis

Target metabonomics was executed by MS, and metabolic fingerprints were assayed by using OPLS-DA, indicating an excellent separation between the control group and fibrotic models, which meant that the data was stable and reliable. These results were further confirmed by volcano. The metabolites in the intestinal contents of UO_2_-exposed rats had significant changes (Differential metabolites and Differential metabolic pathways are presented in Supplementary Materials).

**Figure 4:**
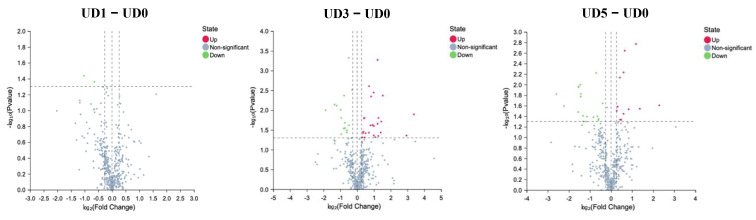
Volcano of different metabolites after UO_2_ exposure between different dose groups.

Compared to the control group, 2-Hydroxybutyric acid, Beta-Alanine, 3,4-Dihydroxymandelic acid, Etiadienic Acid, 12-Dehydrocholic Acid Diacetate, and alpha-Hydroxyisobutyric acid were significantly enriched (*p* < 0.01) while Paroxetine, alpha-linolenic acid, gamma-linolenic acid, L-Lysine, L-Methionine, and L-Leucine were significantly decreased (*p* < 0.01) in the UO_2_-exposed group (Figure 4).

### 3.5. Correlation Analysis Between Gut Microbiota and Metabolite

**Figure 5:**
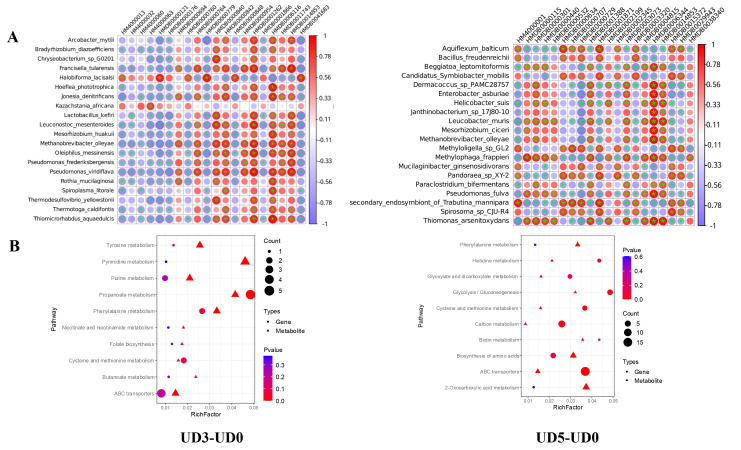
Comprehensive analysis of metabolites and gut microbiota. (**A**). Correlation analysis of metabolite-gut microbiota. UD3-UD0 (**left**), UD5-UD0 (**right**). (**B**). Bubble map of pathway with significant enrichment of differential genes and differential metabolites. UD3-UD0 (**left**), UD5-UD0 (**right**). The “*” representing *p* < 0.05.

In the 2 mg UO_2_ dose group, Folic acid was negatively correlated with *Leuconostoc_mesenteroides* (r = −0.97), 2-Hydroxybutyric acid was negatively correlated with *Chryseobacterium_*sp.*_G0201* (r = −0.91), Maleic acid was negatively correlated with *Pseudomonas_viridiflava* (r = −0.92), Etiadienic acid was negatively correlated with *Pseudomonas_viridiflava* (r = −0.86), 2-Hydroxybutyric acid was negatively correlated with *Arcobacter_mytili* (r = −0.85), Hyocholic acid was negatively correlated with *Methanobrevibacter_olleyae* (r = −0.83), 3, 4-Dihydroxymandelic acid was negatively correlated with *Pseudomonas_viridiflava* (r = −0.82). Beta-Alanine was negatively correlated with *Pseudomonas_viridiflava* (r = −0.65), while Paroxetine was positively correlated with *Lactobacillus_kefiri* (r = 0.79). In the 8 mg UO_2_ dose group, alpha-linolenic acid was negatively correlated with *Stenotrophomonas_*sp.*_YAU14A_MKIMI4_1* (r = −0.85), gamma-linolenic acid was negatively correlated with *secondary_endosymbiont_of_Trabutina_mannipara* (r = −0.80), L-Lysine was negatively correlated with *Erysipelothrix_*sp.*_HDW6C* (r = −0.87), L-Leucine was negatively correlated with *Erysipelothrix_*sp.*_HDW6C* (r = −0.84), L-Methionine was negatively correlated with *Erysipelothrix_*sp.*_HDW6C* (r = −0.83), 3-Hydroxyphenylacetic acid was negatively correlated with *Mucilaginibacter_gotjawali* (r = −0.82), while alpha-linolenic acid was positively correlated with *Sphingomonas_*sp.*_NBWT7* (r = 0.91), gamma-linolenic acid was positively correlated with *Octadecabacter_temperatus* (r = 0.86), and 12-Dehydrocholic Acid Diacetate was positively correlated with *TLactobacillus_zhachilii* (r = 0.89) (Figure 5A). Functionally, the significant metabolic pathways primarily included propanoate metabolism, phenylalanine metabolism, pyrimidine metabolism, and the biosynthesis of amino acids (Figure 5B). In summary, there is a strong correlation between distinct gut microbiota and metabolites under fibrotic pathological conditions.

## 4. Discussion

Hyp is an indispensable amino acid precursor for collagen synthesis and is unique to collagen fibers, which can be used to characterize the degree of fibrosis [34]. Collagen I is the most common fibrillar collagen found in skin, bone, tendons, and other connective tissues. In this study, the contents of Hyp and Collagen I in lung homogenate of rats in the 8 mg dose group were significantly increased at 7 days after exposure (*p* < 0.01), and the expression levels of Hyp and Collagen I in lung homogenate of rats in the 2 mg dose group were significantly increased at 70 days after exposure (*p* < 0.05). This is consistent with reports in animal models of lung fibrosis [35,36]. Histopathology showed that UO_2_ caused certain damage to the lungs of rats. As the dose of UO_2_ increased and the time after exposure extended, the degree of lung damage in rats progressively worsened, culminating in lung fibrosis. In the animal models of uranium-induced lung injury, it was found that uranium aerosols entered the lungs through the respiratory tract and accumulated over time, accompanied by the infiltration of numerous macrophages and inflammatory cells, leading to radiative lesions. These lesions could result in severe parenchymal lymphocyte infiltration, bronchitis, pulmonary abscesses, pulmonary vesicles, and bullae-like lesions [37]. Other studies using U_3_O_8_ suspension injections to assess lung toxicity found that fibroblasts and collagen deposition in the lung interstitium were evident at 15 and 30 days post-exposure [38,39], consistent with the pathological changes observed in this study.

When insoluble uranium oxide particles are inhaled, the lungs serve as a critical target organ due to the particles’ low solubility and prolonged residence time in lung tissue 4. The removal of particulate matter from the respiratory tract after inhalation of uranium aerosol into the body through the respiratory tract involves three different mechanisms. The first mechanism is the mucociliary action of the upper respiratory tract (including the trachea, bronchi, bronchioles, and peripheral bronchioles), which transports deposited particles to the throat, where they are either swallowed into the gastrointestinal tract or expelled through vomiting. The other two mechanisms, dissolution (absorption into the bloodstream) and phagocytosis (clearance by specialized cells), primarily address particles deposited in the deeper respiratory tract (respiratory bronchioles, alveolar ducts, and alveolar sacs). Inhaled uranium, particularly insoluble uranium compounds, is predominantly transported to the throat by the mucociliary escalator and subsequently swallowed into the gastrointestinal tract. Studies have shown that rats exposed to uranium in drinking water (0.2–120 mg/L) exhibited dose-dependent uranium accumulation in bones and kidneys, along with inflammatory cell infiltration in the small intestine, indicating that the gastrointestinal tract is also a target organ for uranium-induced injury [40]. Uranium particles removed by mucocilia can stimulate the gastrointestinal tract. On the one hand, the chemical toxicity of uranium can directly damage the gastrointestinal mucosal epithelial cells, disrupt the structure of the mucus layer, and simultaneously inhibit the repair function of intestinal epithelial cells. This directly alters the living substrate of the gut microbiota (the mucus layer). On the other hand, the mucosal damage caused directly by uranium can activate local gut immunity (such as the activation of macrophages in the lamina propria of the intestinal mucosa), releasing small amounts of pro-inflammatory factors. At this time, if pulmonary inflammation transmits systemic inflammatory signals through the gut–lung axis (such as TNF-α and IL-1β reaching the gut via blood circulation), the two sources of damage will produce a synergistic effect—both exacerbating the disruption of the intestinal barrier and amplifying the over-activation of gut immunity, thereby accelerating the imbalance of microbial structure and metabolic disorders.

TNF-α and IL-1β are key inflammatory factors, and both acute and chronic inflammation are present throughout the entire duration of the rat lung injury model. In this study, as the dose increased or the time after exposure extended, the expression levels of inflammatory factors in the lung homogenate of rats increased. These changes align with experimental results reported in studies on rats [41] and dogs [42,43] exposed to insoluble uranium oxides. The pro-inflammatory factors released during pulmonary inflammation can trigger structural imbalances in the gut microbiota and metabolic disorders through pathways such as damaging the intestinal barrier and interfering with metabolism. These changes, in turn, may reciprocally impact pulmonary inflammation and fibrosis progression via the gut–lung axis, forming a bidirectional regulatory loop. Firstly, TNF-α and IL-1β can reach the intestine via the bloodstream, activating immune cells (such as macrophages and intestinal epithelial cells) within the intestinal mucosa. This activation prompts the release of additional inflammatory mediators, directly damaging the tight junctions between intestinal epithelial cells (e.g., reducing the expression of proteins like occludin and claudin) and compromising intestinal barrier integrity. This impairment leads to a diminished colonization capacity of mucus layer-dependent bacteria (such as *Akkermansia muciniphila*), while bacteria with greater adaptability (such as Bacteroides, which can utilize host metabolites released under inflammatory conditions) proliferate, resulting in an imbalanced microbiota structure. Studies on COVID-19 patients have shown that lung inflammation can lead to altered gut microbiota composition, with harmful bacteria increasing and beneficial ones decreasing. This is linked to increased levels of inflammatory markers and bacterial proteins in the blood [44]. Another study also indicates that plasma concentrations of inflammatory cytokines, chemokines, and tissue damage markers in COVID-19 patients are associated with gut microbiota composition [45]. Secondly, TNF-α and IL-1β can alter the types and concentrations of nutrients available to the gut microbiota (such as carbohydrates and amino acids) by affecting the metabolism of intestinal epithelial cells (e.g., energy metabolism, amino acid transport). This leads to adaptive changes in microbial metabolic pathways (e.g., disruption of propionate synthesis pathways). For example, lower levels of SCFAs and L-isoleucine in stool are associated with disease severity and higher levels of certain plasma inflammatory markers in COVID-19 patients [46].

The gut microbiota of normal rats is dominated by *Firmicutes* and *Bacteroidetes*, and their ratio (F/B) is an important parameter to identify the degree of gut microbiota disturbance. An abnormal increase or decrease in the F/B ratio may indicate gut dysbiosis. In this study, during the process of uranium-induced lung injury in rats, the F/B value of the gut microbiota in the cecum contents decreased, the relative abundance of *Bacteroidetes* increased, the relative abundance of *Verrucomicrobia* decreased, and the relative abundance of AKK and *Akkermansia* decreased after uranium exposure. Inflammatory factors such as TNF-α and IL-1β released during lung injury disrupt the intestinal barrier via the bloodstream. Since AKK’s survival critically depends on an intact mucus layer, barrier damage directly reduces its colonization capacity and abundance. Simultaneously, the direct gastrointestinal effects of uranium (e.g., damaging the intestinal mucosa and inhibiting AKK activity) further exacerbate this reduction. A study found that *Firmicutes* decreased and *Bacteroides* increased in fecal gut microbiota of patients with acute lung injury, and the mouse model also showed a similar imbalance in the proportion of F/B at the phylum level; both patients with acute lung injury and the mouse model showed an imbalance in gut microbiota [47]. Another study indicated that the number of AKK and the F/B value were significantly increased by Scutellaria baicalensis to maintain intestinal homeostasis and alleviated acute lung injury in mice [48]. Some studies have pointed out that the imbalance of gut microbiota may be one of the mechanisms of lung injury induced by heavy metal exposure and thus human health damage [49].

*Akkermansia muciniphila* (AKK) is a mucin-degrading bacterium ubiquitously colonizing in the gut mucosal layer, which belongs to *Verrucomicrobia* [50]. AKK and its derivatives exert a beneficial protective role against diseases such as gut health, pulmonary inflammation, pulmonary fibrosis, etc. On one hand, AKK maintains intestinal barrier integrity by producing short-chain fatty acids (SCFAs, such as propionate). Its reduction leads to SCFA deficiency, further compromising the barrier and allowing endotoxins and pro-inflammatory substances to enter the bloodstream, thereby exacerbating pulmonary inflammation. On the other hand, SCFAs derived from AKK can reach the lungs via the bloodstream, suppressing the release of TNF-α and IL-1β in lung tissue while promoting the proliferation of anti-inflammatory cells (such as regulatory T cells) [51]. The reduction in AKK directly weakens this anti-inflammatory protection in the lungs, accelerating fibrosis. Thus, the decrease in AKK represents a critical intermediate link connecting gut deterioration and lung injury. Consequently, replenishing AKK (through probiotics, prebiotics, or fecal microbiota transplantation) may serve as a targeted therapeutic strategy. A study on 28-day oral gavage of SiO_2_ or Ag nanoparticles in rats found that *Akkermansia*, a genus known for its protective impact on the intestinal barrier, was depleted to hardly detectable levels in Ag nanoparticle-exposed animals [52]. Multiple studies have indicated that AKK supplementation could downregulate the expression of pro-inflammatory cytokines (IL-1, IL-6, and TNF-α) and upregulate the expression of anti-inflammatory cytokines (IL-4, IL-10, and TGF-β), and thus ameliorate the lung damage, highlighting that AKK may be a promising therapeutic option for treating pulmonary inflammation and fibrosis via the gut–lung axis [53,54,55]. Furthermore, the relative abundance of AKK in feces can also be used as a biomarker to predict cancer immunotherapy response [56,57,58]. In lung cancer patients treated with an immune checkpoint blockade, the presence of AKK in the samples may be associated with improved prognosis and enhanced anti-tumor immune cell infiltration [59].

Fecal metabolites are important products of co-metabolism between flora and host, which can not only reflect the state of the gut, but also act as a bridge between symbiotic bacteria and host. Alterations in the gut microbial species and metabolites have been linked to changes in immune response and inflammation as well as the development of lung disease [60,61]. Lipid metabolism plays an important role in many lung functions, and fatty acids are closely related to lung inflammation and can participate in the initiation and resolution of inflammation [62,63]. As an important component of unsaturated fatty acids, linolenic acid is involved in the synthesis of some inflammatory mediators and signaling molecules in the human body. Alpha-linolenic acid possesses potent anti-inflammatory properties, while gamma-linolenic acid provides nutrients to the gut. In this study, compared with the control group, the contents of alpha-linolenic acid and gamma-linolenic acid in the intestinal contents of rats in the 8 mg group were significantly reduced, indicating that lung inflammation had led to the disorder of linolenic acid metabolism. Linolenic acid can be metabolized into anti-inflammatory substances that inhibit the release of TNF-α and IL-1β by pulmonary macrophages, reduce the activation of lung fibroblasts, and simultaneously maintain intestinal barrier integrity. The reduction in AKK may suppress the linolenic acid conversion pathways it participates in (as AKK promotes intestinal lipid absorption and metabolism), whereas the increase in Bacteroides may competitively consume linolenic acid or inhibit its synthesis. Linolenic acid deficiency weakens anti-inflammatory protection in the lungs and impairs intestinal barrier repair capacity. Consequently, endotoxins (e.g., LPS) leak into the bloodstream through the compromised barrier and reach the lungs via circulation, further stimulating pulmonary macrophages to release pro-inflammatory factors. This establishes a positive feedback loop of “intestinal metabolic disorder-exacerbated pulmonary inflammation”. A study showed that alpha-linolenic acid protects against lipopolysaccharide-induced acute lung injury through anti-inflammatory and anti-oxidative pathways. Alpha-linolenic acid pretreatment significantly inhibited the secretion of pro-inflammatory cytokines including TNF-α, IL-6, and IL-1β, and significantly reduced the expression levels of oxidative stress indicators such as myeloperoxidase and malondialdehyde [64]. Another study found that the linoleic acid metabolism pathway exhibited significant metabolic abnormalities at 2 and 4 weeks of pulmonary fibrosis in rats, and the alpha-linolenic acid metabolism pathway significantly affected rat pulmonary fibrosis at 4 weeks [65].

Additionally, *Akkermansia_mucinphila* produces short-chain fatty acids (SCFAs), including acetic acid and propionic acid. Under normal conditions, gut microbiota metabolites (e.g., SCFAs) can suppress pulmonary inflammation via the gut–lung axis (e.g., by inhibiting TNF-α release from pulmonary macrophages). However, after uranium directly damages the gastrointestinal tract, it not only disrupts microbial metabolic function (reducing SCFA production) but may also directly impair intestinal absorption and transport of metabolites (e.g., by damaging transporters in intestinal epithelial cells). This results in weakened “protective signaling” from the gut to the lungs. In this study, compared with the control group, there were significant changes in the propionic acid metabolism pathway of rats in the 2 mg dose group. One study found that metabolic disorders on the “gut–lung” axis exist in mice with LPS-induced acute lung injury, and the content of SCFAs in feces was significantly decreased (*p* < 0.05). After the intervention, the abundance of AKK bacteria, F/B value, acetic acid, and propionic acid contents were increased, which reconstituted intestinal health, increased the concentrations of tryptophan and tyrosine in blood circulation, and then played an anti-inflammatory role to ameliorate acute lung injury [48]. Another study confirmed that Astragalus polysaccharides treatment have a preventive effect on LPS-induced lung injury, which was partly due to the changes in gut microbiota composition in the colon and the resulting increase in the serum concentrations of SCFAs including butyrate and propionate [66]. A recent study also showed that *Akkermansia_mucinphila* effectively alleviated lipopolysaccharides-induced acute lung injury by regulating gut microbiota and supplementing butyrate in mice [67].

## 5. Conclusions

Gut microbiota disturbances and metabolic dysfunction were found in uranium-induced lung injury rats. The underlying mechanism involves the combined effect of pulmonary inflammation and the direct action of uranium on the gastrointestinal tract. This process follows a cascade: “uranium exposure-initiation of lung injury and inflammation-inflammation-induced gut disruption causing microbiota/metabolic dysregulation-gut-derived dysregulation exacerbates pulmonary inflammation and fibrosis via circulatory/immune pathways”. Crucially, the reduction in *Akkermansia* (AKK), proliferation of Bacteroides, and decline in linolenic acid serve as key mediators linking gut–lung injury. Together, these alterations constitute the pathological basis for progression from acute injury to chronic fibrosis. Therefore, AKK supplementation may be a promising treatment option for treating lung disease via the “gut–lung” axis.

## Data Availability

The data presented in this study are available on request from the corresponding author.

## Supplementary Materials

The following supporting information can be downloaded at: https://www.mdpi.com/article/10.3390/metabo15080492/s1, Table S1: Differential metabolites of rat intestinal contents between UD1 and UD0; Table S2: Differential metabolites of rat intestinal contents between UD3 vs. UD0; Table S3: Differential metabolites of rat intestinal contents between UD5 vs. UD0; Table S4: Differential metabolic pathway between UD1 vs. UD0; Table S5: Differential metabolic pathway between UD3 vs. UD0; Table S6: Differential metabolic pathway between UD5 vs. UD0.

## Abbreviations

The following abbreviations are used in this manuscript:

AKK	*Akkermansia muciniphila*
FC	Fold Change
Hyp	Hydroxyproline
ICP-MS	Inductively coupled plasma-Mass Spectrometry
IL-6	Interleukin-6
IL-1β	Interleukin-1β
KEGG	Kyoto Encyclopedia of Genes and Genomes
OPLS-DA	Orthogonal Partial Least-Squares Discrimination Analysis
TGF-β	Transforming Growth Factor-β
TNF-α	Tumor Necrosis Factor-α
UPLC-MS	Ultra Performance Liquid Chromatography- Mass Spectrometry
VIP	Variable Important for the Projection
LEfSe	Linear discriminant analysis Effect Size
