# Supplementary material for: The Impact of Uranium-Induced Pulmonary Fibrosis on Gut Microbiota and Related Metabolites in Rats
Source: Metabolites. 2025 Jul 22;15(8):492. doi: 10.3390/metabo15080492 (PMC12388796; doi:10.3390/metabo15080492)
Supplement: Supplementary file 1 [file metabolites-15-00492-s001.zip › metabolites-3712705-supplementary.pdf]

Table S1 Differential metabolites of rat intestinal contents between UD1 and UD0

Metabolite Name	FC	*P*-value	UD1/UD0	Metabolite ID	KEGG ID
2,6-Pyridinedicarboxylic acid	0.4916	0.036	Down*	HMDB0033161	—
L-Valine	0.6394	0.044	Down*	HMDB0000883	C00183

Table S2 Differential metabolites of rat intestinal contents between UD3 vs UD0

Metabolite Name	FC	*P*-value	UD3/UD0	Metabolite ID	KEGG ID
Hyocholic acid	2.3304	＜0.001	Up***	HMDB0000760	—
Etiadienic Acid	1.6322	0.002	Up**	HM4000032	—
Stearylcarnitine(C18)	1.9768	0.004	Up**	HMDB0000848	—
Maleic acid	2.8795	0.004	Up**	HMDB0000176	C01384
Folic acid	1.7776	0.005	Up**	HMDB0000121	C00504
3,6-Diketocholanic Acid Methyl Ester	10.4226	0.013	Up*	HM4000013	—
4-ANDROSTEN-11beta, 17alpha-DIOL-3-ONE-17beta-CARBOXYLIC ACID	1.3494	0.016	Up*	HM4000063	—
4-Hydroxyphenyl-2-propionic acid	2.4033	0.016	Up*	HMDB0041683	C03080
5beta-ANDROSTAN-3beta-OL-17beta-CARBOXYLIC ACID	1.3233	0.016	Up*	HM4000060	—
Phenyllactic acid	2.6986	0.019	Up*	HMDB0000779	—
3,4-Dihydroxymandelic acid	2.3248	0.022	Up*	HMDB0001866	C05580
N-Formylmethionine	1.8875	0.024	Up*	HMDB0001015	C03145
Beta-Alanine	1.7328	0.024	Up*	HMDB0000056	C00099
L-3-Phenyllactic acid	1.9853	0.025	Up*	HMDB0000748	C05607
2-Hydroxybutyric acid	2.6470	0.037	Up*	HMDB0000008	C05984
Ursocholic Acid	1.6206	0.037	Up*	HMDB0000917	C17644
3,7-Diketocholanic Acid	1.2695	0.038	Up*	HM4000014	—
3,6-Diketocholanic Acid	1.4189	0.038	Up*	HM4000011	—
Cytidine triphosphate	1.9804	0.044	Up*	HMDB0000082	C00063
Adenosine monophosphate	7.6579	0.044	Up*	HMDB0000045	C00020
2-Hydroxy-2-methylbutyric acid	2.3930	0.045	Up*	HMDB0001987	—
23-Nordeoxycholic Acid Diacetate	2.1094	0.048	Up*	HM4000007	—
Heptacosanoic acid	1.2397	0.049	Up*	HMDB0002063	—
Gliquidone	1.3603	0.049	Up*	HMDB0015381	—
3-Hydroxyhippuric	0.7025	＜0.001	Down***	HMDB0006116	—
Salicyluric acid	0.8078	0.003	Down**	HMDB0000840	C07588
Paroxetine	0.5827	0.004	Down**	HMDB0014853	C07415
Quinaldic acid	0.3936	0.007	Down**	HMDB0000842	C06325
Dehydrolithocholic acid	0.4265	0.008	Down**	HMDB0001262	C01835
2-Phenylpropionic acid	0.5091	0.010	Down**	HMDB0011743	—
Ferulic acid	0.2718	0.010	Down*	HMDB0000954	C01494
Hydrocinnamic acid	0.5178	0.016	Down*	HMDB0000764	C05629
2-hydroxyglutaric acid	0.6033	0.021	Down*	HMDB0000694	C03196
Alanylglutamine	0.7123	0.024	Down*	HMDB0028685	—
Uridine 5'-monophosphate	0.6067	0.028	Down*	HMDB0000288	C00105
Melibiose	0.5746	0.029	Down*	HMDB0000048	—
2,6-Pyridinedicarboxylic acid	0.6560	0.032	Down*	HMDB0033161	—
Xanthosine	0.6528	0.037	Down*	HMDB0000299	C01762
Nonanoic acid	0.5176	0.040	Down*	HMDB0000847	C01601

Table S3 Differential metabolites of rat intestinal contents between UD5 vs UD0

Metabolite Name	FC	*P*-value	UD5/UD0	Metabolite ID	KEGG ID
12-Dehydrocholic Acid Diacetate	2.2683	0.002	Up**	HM4000001	—
Clomipramine	1.5699	0.002	Up**	HMDB0015372	C06918
alpha-Hydroxyisobutyric acid	1.5089	0.006	Up**	HMDB0000729	C21297
4-Hydroxyphenylpyruvic acid	1.3385	0.007	Up**	HMDB0000707	C01179
Epicatechin	4.8876	0.025	Up*	HMDB0001871	C09727
Indole-3-carboxylic acid	1.2435	0.026	Up*	HMDB0003320	C19837
Citraconic acid	2.5755	0.029	Up*	HMDB0000634	C02226
Arbutin	1.7859	0.029	Up*	HMDB0029943	C06186
Heneicosanoic acid	1.2088	0.031	Up*	HMDB0002345	—
L-Thyronine	1.5043	0.036	Up*	HMDB0000667	—
Epigallocatechin gallate	1.3447	0.046	Up*	HMDB0003153	C09731
Etodolac	1.3937	0.047	Up*	HMDB0014887	C06991
Paroxetine	0.6116	0.006	Down**	HMDB0014853	C07415
N-(p-Coumaroyl)serotonin	0.3636	0.010	Down*	HMDB0038340	—
4-Hydroxy-3-methylbenzoic acid	0.3461	0.011	Down*	HMDB0004815	C21167
3-Hydroxyphenylacetic acid	0.3416	0.011	Down*	HMDB0000440	C05593
Alpha-Linolenic acid	0.3717	0.015	Down*	HMDB0001388	C06427
Alpha-N-Phenylacetyl-L-glutamine	0.1660	0.015	Down*	HMDB0006344	C04148
gamma-Linolenic acid	0.3691	0.017	Down*	HMDB0003073	C06426
Urocanic acid	0.3691	0.017	Down*	HMDB0000301	C00785
Acetylglycine	0.7599	0.023	Down*	HMDB0000532	—
Glycolic acid	0.2128	0.026	Down*	HMDB0000115	C00160
Desaminotyrosine	0.3395	0.033	Down*	HMDB0002199	C01744
Ferulic acid	0.3973	0.039	Down*	HMDB0000954	C01494
L-Kynurenine	0.6376	0.040	Down*	HMDB0000684	C00328
L-Lysine	0.4544	0.040	Down*	HMDB0000182	C00047
2,6-Pyridinedicarboxylic acid	0.5604	0.042	Down*	HMDB0033161	—
L-Methionine	0.6678	0.045	Down*	HMDB0000696	C00073
L-Leucine	0.6589	0.047	Down*	HMDB0000687	C00123
1-Methylhistidine	0.4008	0.050	Down*	HMDB0000001	—

Table S4 Differential metabolic pathway between UD1 vs UD0

Pathway	Total	Metabolite Number	*P*-value	State	Metabolites
Valine, leucine and isoleucine biosynthesis	23	1	0.001	Down**	L-Valine
Pantothenate and CoA biosynthesis	28	1	0.002	Down**	L-Valine
Mineral absorption	29	1	0.002	Down**	L-Valine
Central carbon metabolism in cancer	37	1	0.002	Down**	L-Valine
Valine, leucine and isoleucine degradation	42	1	0.002	Down**	L-Valine
Protein digestion and absorption	47	1	0.003	Down**	L-Valine
Aminoacyl-tRNA biosynthesis	52	1	0.003	Down**	L-Valine
Biosynthesis of amino acids	128	1	0.007	Down**	L-Valine
2-Oxocarboxylic acid metabolism	134	1	0.007	Down**	L-Valine
ABC transporters	137	1	0.007	Down**	L-Valine

Table S5 Differential metabolic pathway between UD3 vs UD0

Pathway	Total	Metabolite Number	*P*-value	State	Metabolites
Pyrimidine metabolism	65	3	＜0.001	Up****	Beta-Alanine; Cytidine triphosphate; Uridine 5'-monophosphate
Antifolate resistance	17	2	＜0.001	Up***	Adenosine monophosphate; Folic acid
Propanoate metabolism	48	2	0.001	Up**	2-Hydroxybutyric acid; Beta-Alanine
Phenylalanine metabolism	60	2	0.002	Down**	L-3-Phenyllactic acid; Hydrocinnamic acid
Tyrosine metabolism	78	2	0.003	Up**	Maleic acid; 3,4-Dihydroxymandelic acid
Mannose type O-glycan biosynthesis	4	1	0.004	Up**	Cytidine triphosphate
mTOR signaling pathway	4	1	0.004	Up**	Adenosine monophosphate
PI3K-Akt signaling pathway	4	1	0.004	Up**	Adenosine monophosphate
Purine metabolism	95	2	0.005	Down**	Adenosine monophosphate; Xanthosine
FoxO signaling pathway	5	1	0.006	Up**	Adenosine monophosphate

Table S6 Differential metabolic pathway between UD5 vs UD0

Pathway	Total	Metabolite Number	*P*-value	State	Metabolites
2-Oxocarboxylic acid metabolism	134	5	＜0.001	Down****	L-Lysine; Citraconic acid; L-Leucine; L-Methionine; 4-Hydroxyphenylpyruvic acid
Biosynthesis of amino acids	128	4	＜0.001	Down****	L-Lysine; L-Leucine; L-Methionine; 4-Hydroxyphenylpyruvic acid
Protein digestion and absorption	47	3	＜0.001	Down****	L-Lysine; L-Leucine; L-Methionine
Aminoacyl-tRNA biosynthesis	52	3	＜0.001	Down****	L-Lysine; L-Leucine; L-Methionine
Valine, leucine and isoleucine biosynthesis	23	2	＜0.001	Down***	Citraconic acid; L-Leucine
Mineral absorption	29	2	＜0.001	Down***	L-Leucine; L-Methionine
Central carbon metabolism in cancer	37	2	＜0.001	Down***	L-Leucine; L-Methionine

Pathway	Total	Metabolite Number	*P*-value	State	Metabolites
Phenylalanine metabolism	60	2	0.002	Down**	3-Hydroxyphenylacetic acid; Alpha-N-Phenylacetyl-L-glutamine
Biosynthesis of unsaturated fatty acids	74	2	0.004	Down**	Alpha-Linolenic acid; gamma-Linolenic acid
Tyrosine metabolism	78	2	0.004	Down**	3-Hydroxyphenylacetic acid; 4-Hydroxyphenylpyruvic acid
